# Endovascular treatment of true visceral artery aneurysms: a decade of experience and key outcomes from a high-volume single center

**DOI:** 10.1186/s42155-026-00664-0

**Published:** 2026-02-28

**Authors:** Laura Maria Cacioppa, Pietro Boscarato, Tommaso Valeri, Francesco Mariotti, Alessandra Bruno, Nicolò Rossini, Giangabriele Francavilla, Alice Aste, Olisea Di Lello, Marzia Rosati, Alessandro Felicioli, Vincenzo Vento, Emanuele Gatta, Laura Giantomassi, Enrico Paci, Roberto Candelari, Chiara Floridi

**Affiliations:** 1 Division of Interventional Radiology, Department of Radiological Sciences, University Hospital “Azienda Ospedaliero Universitaria Delle Marche”, Ancona, 60126 Italy; 2 Department of Clinical, Special and Dental Sciences, University Politecnica Delle Marche, Ancona, 60126 Italy; 3 Division of Radiology, Department of Radiological Sciences, University Hospital “Azienda Ospedaliero Universitaria Delle Marche”, Ancona, 60126 Italy; 4 Vascular and Endovascular Surgery Unit, University Hospital “Azienda Ospedaliero Universitaria Delle Marche”, Ancona, 60126 Italy; 5 Vascular Medicine Unit, University Hospital “Azienda Ospedaliero Universitaria Delle Marche”, Ancona, 60126 Italy; 6 Diagnostic Imaging, Clinical and Interventional Radiology, IRCCS INRCA, Ancona, Italy

**Keywords:** Endovascular aneurysm repair, Patient selection, Endovascular procedures, Angiography, Digital subtraction, Computed tomography angiography, Radiology, Interventional, Follow-up studies

## Abstract

**Background:**

Endovascular approach has emerged as the first-choice treatment of visceral artery aneurysms (VAAs). However, most published series include heterogeneous populations combining true aneurysms and pseudoaneurysms. Our single-center retrospective study aimed to evaluate a large and homogenous series of true VAAs treated endovascularly. Demographic, clinical and procedural details of patients with VAAs treated at our Interventional Radiology unit from January 2014 to January 2025 were collected in a dedicated database. Intraprocedural and perioperative outcomes included technical success, complications, mortality and need for reintervention. At long-term follow-up, overall survival, aneurysm-related complications, freedom from reintervention, and aneurysmal sac reperfusion were evaluated.

**Results:**

Ninety-four consecutive patients (41 men; median age: 67.3 years; range 29–98 years) with 97 VAAs were included in the study. VAAs had a median diameter of 2.93 cm (range 6–9.2 cm). Treatment strategies included sac packing in 45 VAAs, sandwich technique in 35, covered stent placement in 13, afferent artery occlusion in 5 and stent-assisted coiling in 4 cases. Technical success rate was 100% with no perioperative deaths. Perioperative major complication and reintervention rates were 5.2% and 2.1%, respectively. At a mean follow-up of 44.7 ± 28.9 months, overall survival, freedom from aneurysm-related complications, and freedom from reintervention were 89.4%, 92.8%, and 93.8%, respectively. Aneurysm sac reperfusion was observed in 8(8.2%) cases. No aneurysm-related deaths occurred.

**Conclusion:**

Endovascular approach is a safe and effective first-line option in patients with true VAAs, with notable results in terms of complication and freedom from reintervention rates and an excellent long-term overall survival.

**Graphical Abstract:**

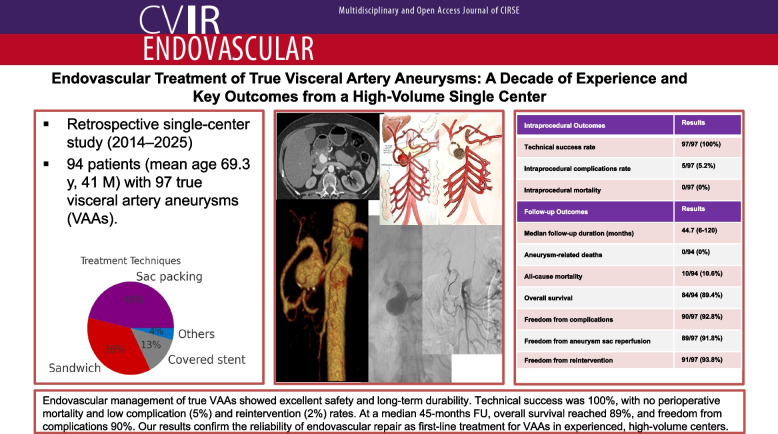

## Introduction

True visceral artery aneurysms (VAAs) are rare but potentially life-threatening lesions, with an incidence ranging from 0.01% to 0.2% and accounting for less than 5% of all intra-abdominal aneurysms [1, 2]. The etiology of VAAs is predominantly atherosclerotic; however, non-atherosclerotic causes including connective-tissue disorders (e.g. Ehlers–Danlos and Marfan syndromes), fibromuscular dysplasia, arterial vasculitis (e.g. Takayasu arteritis) and high-flow conditions such as portal hypertension or pregnancy may be particularly relevant in younger patients [3].

True VAAs must be clearly distinguished from false (pseudo)aneurysms, as they represent distinct pathological entities with different etiologies, natural histories, and therapeutic implications.

True aneurysms involve all the arterial wall layers, whereas pseudoaneurysms result from focal inner-wall breach secondary to trauma, infection, pancreatitis, or iatrogenic causes [4, 5]. This distinction is clinically crucial, since pseudoaneurysms typically warrant immediate treatment irrespective of size, whereas true VAAs require a more individualized management, according to aneurysm diameter, growth rate, anatomical site, and clinical presentation [2, 3, 10].

Although most VAAs remain asymptomatic and are discovered incidentally, rupture occurs in approximately 3% of cases and may be the picture of onset, often associated with high mortality rates [3, 6–8].

Despite a slight variability, current treatment indications are primarily based on size thresholds, with additional consideration given to growth dynamics, arterial segment involved, and additional patient-related factors such as childbearing age [2, 3, 9–11]. While a 2 cm treatment threshold was historically reported, recent guidelines recommend intervention for splenic and renal artery VAAs > 3 cm, although this remains debated [2, 3, 9–11]. Gastric, gastroepiploic, pancreaticoduodenal, and mesenteric aneurysms warrant treatment regardless of size due to their high rupture risk, as well as any symptomatic or rapid growing (> 0.5 cm/year), and those detected in women of childbearing age [2, 3, 11].

Over the last decade, endovascular treatment has emerged as the preferred therapeutic option for anatomically suitable VAAs, offering a minimally invasive strategy associated with lower perioperative morbidity and mortality compared with surgery [12–16]. However, most available evidence results from heterogeneous series that combine true and pseudoaneurysms, limiting the interpretation of outcomes specific to true VAAs. The present study reports a decade-long, high-volume single-center experience focusing exclusively on true VAAs endovascular treatment. The aim was provide a robust real-world evidence on early and long-term outcomes assessing safety, efficacy, durability, and key clinical endpoints.

## Materials and methods

### Study population and data collection

Between January 2014 and January 2025, a consecutive series of 94 patients with 97 true VAAs submitted to endovascular treatment at the Interventional Radiology Unit of our Institution were retrospectively included in this study. The study was conducted in accordance with the Declaration of Helsinki and approved by our Institutional Review Board. True VAAs were diagnosed on preprocedural CTA as a focal, permanent arterial dilatation involving all layers of the vessel wall, with preservation of wall continuity and without evidence of contrast extravasation, perivascular hematoma, or arterial wall disruption. Lesions showing signs of focal wall breach, contained rupture, active bleeding, or pseudoaneurysm were systematically excluded.

Informed consent was obtained from all subjects involved in the study. Files and images were extracted from the three single RIS (Radiology Information) and PACS (Picture Archiving and Communication) systems. Clinical data, including length of hospital stay, and perioperative details were obtained from digital medical records.

Follow-up data were obtained via institutional PACS or, when unavailable, by telephone interviews with patients or their referring physicians.

Demographic variables, clinical presentation (asymptomatic, symptomatic without rupture, or ruptured aneurysm), comorbidities including hypertension, type 2 diabetes mellitus, coronary artery disease, smoking history, chronic obstructive pulmonary disease (COPD), and dyslipidemia, as well as anatomical features (including the presence of associated aortic, iliac, femoral, or popliteal aneurysms), aneurysm location (splenic, renal, hepatic, gastroduodenal, pancreaticoduodenal, celiac trunk, or superior mesenteric arteries), and the presence of multiple aneurysms were collected in a dedicated database. Elective treatment was performed in asymptomatic patients or in symptomatic patients without imaging evidence of rupture whereas emergency treatment was performed in cases of aneurysm rupture or imaging signs of active bleeding.

The analysis also included procedural variables such as preoperative aneurysm diameter, procedural strategy (coil embolization with sandwich, sac packing, afferent-only, or efferent-only approach, covered stenting or stent-assisted coiling, alternative techniques such as flow-diverters and plugs), technical details such as number of coils or stents deployed were also registered. Outcome-related variables included technical success, intra-procedural complications, length of hospital stay, perioperative mortality, complication and reintervention rates, and long-term follow-up data.

### Inclusion criteria and indications for treatment

Inclusion criteria included the presence of at least one true VAA with endovascular treatment performed at our institution, and availability of complete pre- and post-procedural imaging and clinical data. Treatment indications included symptomatic aneurysms regardless of size, and asymptomatic aneurysms with a diameter > 2 cm. Selected cases of splenic and renal aneurysms between 2 and 3 cm as well as those with saccular morphology, small-vessel involvement, or aneurysms < 2 cm (e.g., in women of childbearing age or with documented rapid growth) were discussed within a multidisciplinary team including Vascular Surgeons. These criteria were consistent with the most recent guidelines from the Society for Vascular Surgery (SVS) and the joint recommendations of the Italian Societies of Vascular Surgery (SICVE) and Interventional Radiology (SIRM) [3, 9].

Exclusion criteria included all pseudoaneurysms and other non-aneurysmal vascular lesions, contraindications to iodinated contrast agents, known hypersensitivity to endovascular devices, and absent, incomplete, or lost patient follow-up.

### Preoperative imaging

All patients underwent preprocedural contrast-enhanced computed tomography angiography (CTA), performed with either single- or dual-energy CT scanners, using a standardized protocol that included non-contrast, arterial (20–30 s post-contrast), and venous (approximately 45 s) phases [17]. The protocol included the entire thoracoabdominal aorta and visceral vessels, ensuring thorough assessment for precise treatment planning. For patients imaged at our institution with dual-energy CT scanner (Siemens Somatom Force, Siemens Healthcare, Forchheim, Germany), high-resolution axial acquisitions (0.625 mm), multiplanar reconstructions (MPR), and 3D volume-rendering images were systematically obtained for treatment planning. Imaging assessment focused on aneurysm morphology and size, afferent and efferent vessels diameter, neck characteristics, proximity to arterial bifurcations, collateral circulation, and signs of organ ischemia.

### Procedural technique and strategy

All the procedures were performed in the fully equipped angiosuites of our Institution: Philips Allura Xper FD 20, Philips Azurion 7 M20 (Philips Healthcare, Best, Netherlands), and Siemens Axiom Artis Zee (Siemens AG, Forchheim, Germany) systems. Treatments were performed by six interventional radiologists with ≥ 5 years of experience in vascular Interventional Radiology. A tailored treatment strategy was adopted according to aneurysm characteristics, vessel tortuosity, presence of landing zones, collateral circulation, technical feasibility, and operator expertise. Patients were prepared with trichotomy, skin disinfection and sterile draping. All procedures were performed under local anesthesia. Intravenous sedation or analgesia were administered if needed. Arterial access was predominantly achieved via a unilateral transfemoral approach, with simultaneous or exclusive left brachial access reserved for marked vascular tortuosities. Peri-procedural anticoagulation consisted of 5,000 IU of intra-arterial heparin. After vascular access and selective angiography using a 4 F hydrophilic diagnostic catheter, super-selective catheterizations were performed using 1.9 to 2.7 Fr microcatheter systems. In saccular aneurysms, or when preservation of collaterals arising from the aneurysmal sac was not required, the “sac packing” technique was preferred—particularly in cases with a narrow aneurysmal neck. In contrast, for fusiform aneurysms, the “isolation” or “sandwich” technique (coil embolization of both afferent and efferent vessels), or single inflow or outflow vessel embolization, was employed based on anatomical feasibility. Pushable or detachable platinum microcoils (0.035" or 0.018") of varying numbers and sizes were delivered through microcatheters following super-selective cannulation of the target vessel. In fusiform aneurysms with adequate proximal and distal landing zones, particularly in cases of high risk of organ ischemia, placement of balloon-expandable or self-expandable covered stents was preferred. Balloon dilation of both sealing zones was routinely performed to optimize stent apposition. In complex lesions near vascular bifurcations or requiring major collateral branches preservation, stent-assisted coiling or ballon-assisted coiling and flow-diverting stents were considered. Hemostasis was achieved by manual compression at puncture site. Postoperatively, a single antiplatelet agent was administered, whereas in cases involving stent placement, dual antiplatelet therapy was recommended for at least one year.

### Follow-up protocol

In accordance with current recommendations [3, 9, 10], all patients were submitted to a standard peri- and post-operative surveillance protocol including clinical assessment and contrast-enhanced CTA (Siemens Somatom Force, Siemens Healthcare, Forchheim, Germany) or MR angiography (MRA) at 1, 6, and 12 months, and yearly thereafter. Postoperative CTA follow-up was performed using the same triphasic protocol adopted for preoperative imaging; when feasible, a dual-energy acquisition protocol was employed to minimize artifacts from metallic devices and improve image quality. Imaging studies were systematically reviewed to assess aneurysm size, complications including sac reperfusion, endoleaks, stent-related issues, aneurysm sac changes, and the presence of organ ischemia.

### Definitions and outcomes

Intraprocedural outcomes were evaluated in terms of technical success rate, intraprocedural complication rate, and intraprocedural mortality. Technical success was defined as complete disappearance of aneurysmal flow in patients treated with embolization, and as complete exclusion of the aneurysm in those undergoing covered stent placement, as confirmed by completion angiography at the end of the procedure. Intraprocedural mortality was defined as death occurring during the endovascular procedure. Intraprocedural complications included any adverse events occurring during the intervention, such as vessel rupture, distal embolization, device migration, or the need for surgical conversion.

Perioperative outcomes, defined as events occurring during the hospital stay and within 30 days after the procedure, included length of hospital stay, complication rates, mortality, and the need for open or endovascular reintervention. Perioperative complications included any adverse events occurring during the hospital stay or within 30 days after the procedure. All procedure-related complications were classified according to the CVIR classification system [18].

Follow-up outcomes were evaluated in terms of survival, the need for open or endovascular reintervention, freedom from aneurysm-related complications, and aneurysm sac reperfusion or recanalization. *Aneurysm-related complications* were defined as any adverse events directly involving the treated aneurysm or its parent vessel, including aneurysm rupture, stent thrombosis or migration, distal embolization, or device-related failures requiring intervention. Aneurysm sac reperfusion or recanalization was defined as the persistence or reappearance of blood flow within the aneurysmal sac after embolization or stent placement, including the occurrence of type II endoleak, indicating incomplete exclusion or loss of aneurysm occlusion over time. This finding, detected on follow-up imaging, was considered a marker of procedural durability.

### Statistical analysis

The results of univariate analysis were tested for significance using Chi-square test. Multivariate analysis was performed using binary logistic regression. Factors with *p* < 0.05 at univariate analysis were included in multivariate analysis.

All statistical analyses were performed using SPSS version 22.0 (IBM Corp., Armonk, NY, USA). A *p* value < 0.05 was considered statistically significant.

Continuous variables were expressed as mean ± standard deviation (SD) and median with range, as appropriate. Categorical variables were expressed as absolute numbers and percentages.

The outcomes analyzed included technical success, intraprocedural and perioperative complications, perioperative mortality, aneurysm sac reperfusion or recanalization, the need for open or endovascular reintervention, and overall survival during follow-up.

Early and follow-up results were analyzed using Chi-square (χ^2^) or Fisher’s exact test, as appropriate, for categorical variables. Continuous variables were compared using Student’s *t* test or Mann–Whitney *U* test, as applicable. Follow-up outcomes, including overall survival and freedom from aneurysm-related complications or reintervention, were evaluated using life-table analysis and Kaplan–Meier survival curves.

## Results

### Demographics, anatomical, and clinical findings

A total of 97 true VAAs in 94 patients (41 males, 43.6%) were collected and included in the final analysis. The median patient age was 67.3 years (range 29–98 years). The majority of aneurysms were located in the splenic artery (64/97, 66%), followed by the pancreaticoduodenal artery (9/97, 9.3%), renal artery (8/97, 8.2%), hepatic artery (7/97, 7.2%), celiac trunk (7/97, 7.2%), gastroduodenal artery (1/97, 1%), and ileo-colic branch of superior mesenteric artery (1/97, 1%).

The median preoperative aneurysm diameter was 2.93 cm (range 6–9.2 cm). Multiple VAAs were observed in 23 patients (24.5%). Patient cardiovascular comorbidities and vascular risk factors included arterial hypertension (58/94, 61.7%), type II diabetes (19/94, 20.2%), coronary artery disease (17/94, 18.1%), chronic obstructive pulmonary disease (COPD: 12/94, 12.8%), dyslipidemia (35/94, 37.2%), and a history of smoking (31/94, 33%). Concomitant aneurysms in other major vascular territories were observed in a minority of patients, including aortic aneurysm > 4.5 cm (5/94, 5.3%), iliac artery aneurysm (4/94, 4.3%), popliteal artery aneurysm (8/94, 8.5%), and femoral artery aneurysm (4/94, 4.3%).

Most VAAs were identified incidentally during imaging performed for unrelated clinical indications (77/97, 79.4%). Symptomatic aneurysms were present in 20/97 patients (20.6%), with the most common initial manifestations including nonspecific abdominal pain or discomfort, nausea and vomiting, and flank pain or hematuria in renal artery VAAs. At presentation, 16/97 aneurysms (16.5%) were ruptured, either actively, imminently, or in a contained state.

Demographic characteristics of the study population, arterial sites affected by VAAs, associated comorbidities, vascular risk factors, and clinical presentations are summarized in Table 1.

**Table 1:** Demographic characteristics of the study population (*n* = 94 patients; *n* = 97 VAAs), arterial territories of visceral artery aneurysms, comorbidities, vascular risk factors, and clinical presentation of VAAs

Demographics	
• Age (yrs)	67.3
• Range	(29–98)
• Sex (male/female)	41/53
Anatomical characteristics	
Arterial site	n (%)
• Splenic artery	64 (66%)
• Renal artery	8 (8.2%)
• Hepatic artery	7 (7.2%)
• Gastroduodenal artery	1 (1%)
• Pancreaticoduodenal artery	9 (9.3%)
• Celiac trunk	7 (7.2%)
• Superior mesenteric artery (SMA)	1 (1%)
Median preoperative diameter (cm)	2.93 (0.6–9.2)
Multiple lesions	23
Vascular risk factors and comorbidities	n (%)
• Arterial hypertension	58 (63%)
• Type II diabetes	19 (20.7%)
• Coronary heart disease	17 (18.5%)
• History of smoking	31 (33.7%)
• COPD*	12 (13.04%)
• Hyperlipidemia	35 (38.04%)
• Aortic aneurysm(> 4.5 cm)	5 (3.43%)
• Iliac artery aneurysm	4 (4.3%)
• Popliteal artery aneurysm	8 (8.7%)
• Femoral artery aneurysm	4 (4.3%)
Clinical presentation	n (%)
• Asymptomatic/incidentally detected	77 (79.4%)
• Symptomatic (e.g., pain or compression symptoms)	20 (20.6%)
• Ruptured VAA	16 (16.5%)

^*^Chronic obstructive pulmonary disease

### Procedural details

Endovascular treatment of VAAs was successfully performed in 100% of cases. The most frequently employed techniques were sac-packing in 45 VAAs and sandwich technique in 35. Afferent arterial branch embolization was performed in 5 cases. A covered stent was employed in 13 cases, particularly in aneurysms with suitable landing zones. Stent- or balloon-assisted coiling were adopted in 4 anatomically complex lesions. Endovascular plugs were employed in 27 aneurysms. In some cases, techniques were used in combination.

**Fig. 1:**
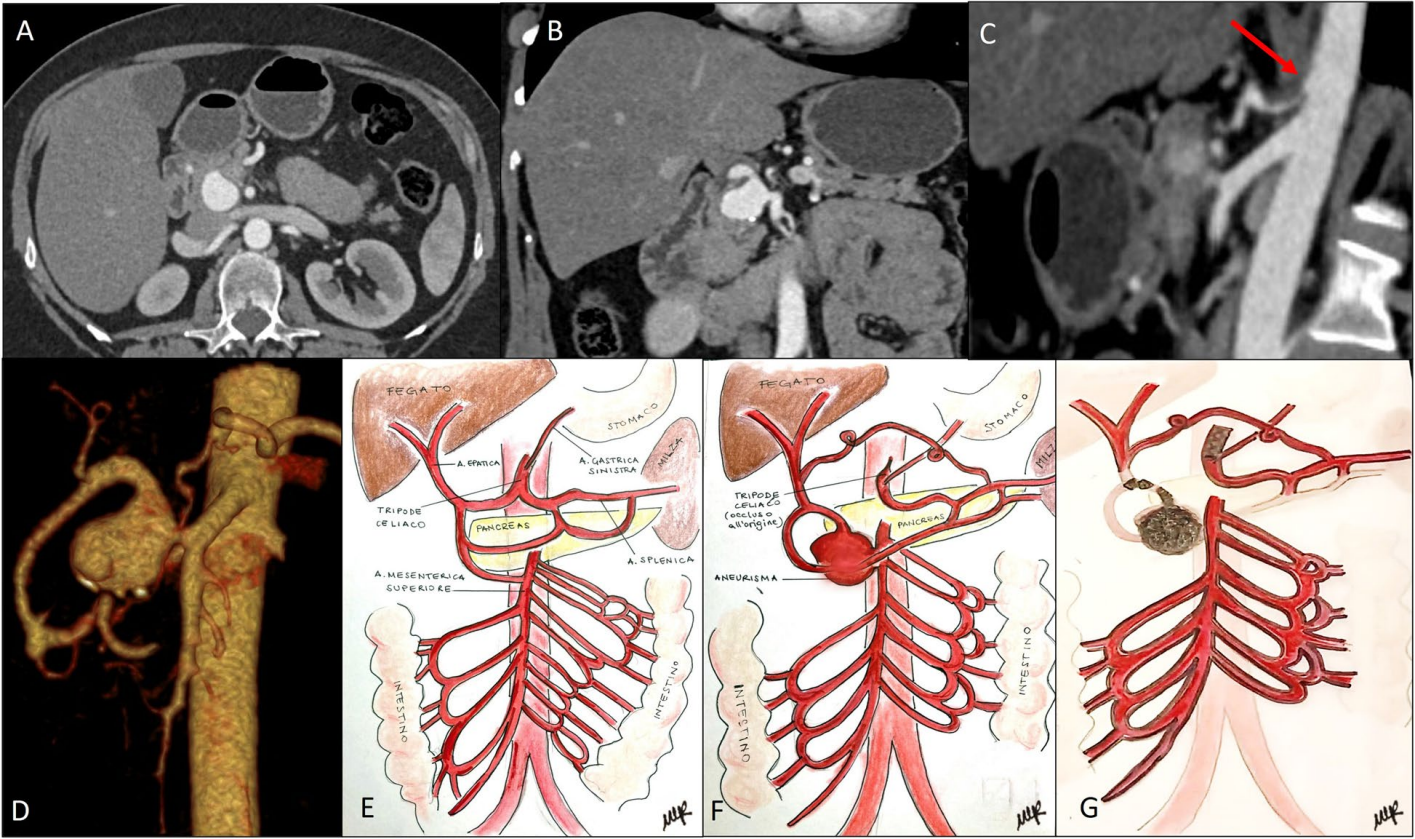
Abdominal computed tomography angiography (CTA) of a 51-year-old woman demonstrating a 2.6 cm superior mesenteric artery (SMA) aneurysm in axial (**A**) and coronal (**B**) planes. Sagittal reconstruction (**C**) shows near-complete occlusion of the celiac trunk (red arrow). Volume-rendered reconstruction (**D**) illustrates the aneurysm arising from the SMA, which distally gives rise to the proper hepatic artery and a hypertrophic pancreaticoduodenal arcade (PDA) replacing the celiac axis and, through flow reversal, supplying the splenic artery. Illustrative anatomical sketches (**E**–**G**), used as explanatory material during the informed consent process, show the normal visceral arterial anatomy (**E**), the altered anatomy with the VAA (**F**), and the post-procedural configuration following aneurysm embolization and celiac trunk stenting (**G**)

**Fig. 2:**
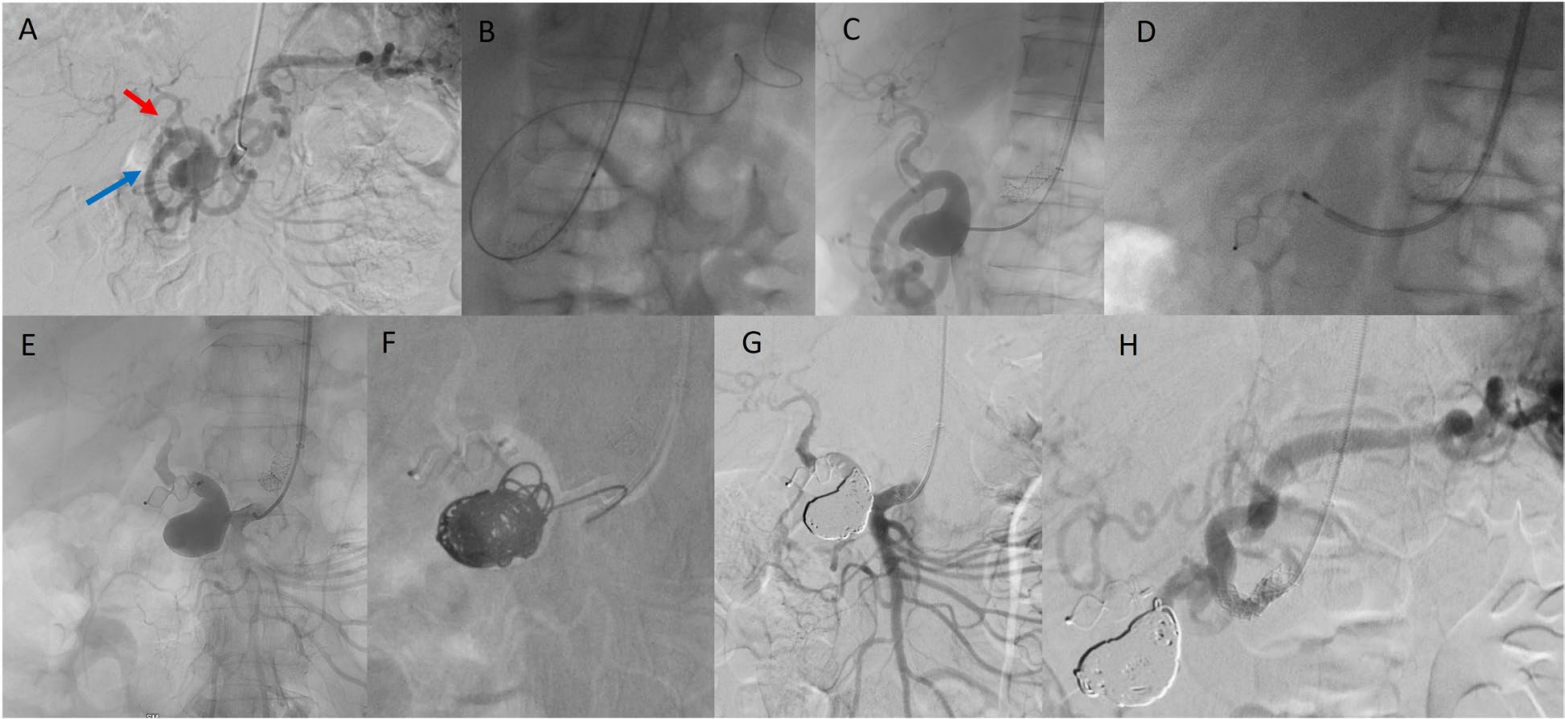
After an adequate procedural planning and multidisciplinary discussion, the VAA illustrated in Fig. 1 was submitted to endovascular treatment within a few days from CTA. Selective superior mesenteric artery (SMA) angiography (**A**) confirms CTA findings, demonstrating a SMA aneurysm with distal bifurcation into the proper hepatic artery (red arrow) and a hypertrophic pancreaticoduodenal arcade (PDA-blue arrow), with retrograde opacification of the splenic artery due to flow reversal through the PDA. Subsequent angioplasty and stenting of the celiac trunk (**B**, **C**) led to the disappearance of splenic artery opacification. A vascular plug was then deployed within the PDA arcade to exclude the efferent branch of the aneurysm. The aneurysmal sac was then excluded by sac embolization technique (**D**–**F**). Final angiograms from the SMA (**G**) and celiac trunk (**H**) confirmed complete aneurysm exclusion with preserved hepatic, splenic, and intestinal perfusion

The mean number of coils deployed per procedure was 4.66 ± 3.29. A mean of 1.2 stents were deployed per patient, with an average stent diameter of 5.3 mm. No high-flow intraprocedural endoleaks requiring additional covered stents were observed. An illustrative case of a superior mesenteric artery aneurysm associated with celiac trunk occlusion, treated with combined stenting and embolization, is presented in Figs. 1 and 2.

Intraprocedural complications occurred in 5/97 cases (5.7%). In one case, distal migration of one coil was observed and successfully retrieved using a goose-neck snare. Two additional patients, both with splenic artery aneurysms, developed acute central abdominal pain requiring monitoring and medical management. One of these cases involved a splenic artery rupture, which was effectively treated with the prompt deployment of a covered stent. In another patient, a limited dissection distal to the stent was noted without affecting downstream flow. No intraprocedural deaths occurred. Procedural details, including the endovascular techniques applied according to the treated arterial territory, were summarized in Table 2.

**Table 2:** Embolization technique by arterial territory

Involved vessel	Coil embolization			Covered Stenting	Stent/balloon-assisted coiling	Endovascular plugs
	Sac-packing	Sandwich	Afferent occlusion			
• Splenic artery	30	26	3	5	0	22
• Renal artery	4	0	0	4	4	0
• Hepatic artery	5	0	1	1	0	2
• Gastroduodenal artery	1	0	0	0	0	0
• Left gastric artery	1	0	0	0	0	0
• Pancreaticoduodenal artery	2	7	0	0	0	3
• Celiac trunk	2	1	1	3	0	0
• Superior Mesenteric artery (ileo-colic branch)	0	1	0	0	0	0

Distribution of embolization techniques including coil embolization (“sac packing” technique, “sandwich” or “isolation” technique and single afferent or efferent vessel embolization), covered stenting, stent- or balloon-assisted coiling, and employment of endovascular plugs according to the arterial district treated. In some cases, techniques were used in combination

### Perioperative outcomes

No deaths were recorded during hospitalization or within the first 30 days after the procedure. Perioperative complications were observed in 5/97 (5.2%) cases. These included three case of extensive and symptomatic splenic ischemia, in two cases requiring splenectomy 7 and 10 days post-procedure, and in one case requiring clinical monitoring and management of pain symptoms; one case of massive bilateral pleural effusion, and one case of significant perisplenic fluid collection successfully managed with percutaneous drainage. All these cases required prolonged hospitalization. As expected consequence of endovascular embolization, a total of 13/97 (13.4%) minor complications consisting in uncomplicated segmental organ ischemia were also observed, 11 involving the spleen and 2 the kidney, all asymptomatic or mildly symptomatic, self-limiting, and managed conservatively. Overall, the mean hospital stay was 3.3 ± 1.8 days. There were no hospital readmissions and no other 30-day procedure-related major complications. Overall, the perioperative reintervention rate was 2.1%.

Intraprocedural and perioperative outcomes, including technical success, mortality, complications, reintervention rate and length of hospital stay, were summarized in Table 3.

**Table 3:** Summary of intraprocedural, perioperative, and follow-up outcomes of the study cohort (*n* = 94 patients; *n *= 97 VAAs)

Intraprocedural outcomes	Result
• Technical success rate	97/97 (100%)
• Intraprocedural complication rate	5/97 (5.2%)
• Intraprocedural mortality	0/97 (0%)
Perioperative Outcomes	Result
• Mean hospital stay (days)	3.3±1.83
• Perioperative mortality	0/97 (0%)
• Perioperative major complications*	5/97 (5.2%)
• Perioperative minor complications**	13/97 (13.4%)
• Perioperative reintervention rate	2/97 (2.1%)
Follow-up Outcomes	Result
• Median follow-up duration (months)	44.7(6–120)
• Aneurysm-related deaths	0/94 (0%)
• All-cause mortality	10/94 (10.6%)
• Overall survival	84/94 (89.4%)
• Freedom from aneurysm-related complications	90/97 (92.8%)
• Freedom from aneurysm sac reperfusion or recanalization	89/97 (91.8%)
• Freedom from reintervention	91/97 (93.8%)

Procedure-related complications were classified as defined by the CVIR classification system

*Procedure-related adverse events occurring during the hospital stay or within 30 days after the procedure, which required additional invasive treatment, resulted in prolonged hospitalization, caused permanent morbidity, or were potentially life-threatening

**Asymptomatic or mildly symptomatic segmental organ ischemias, which were self-limiting and did not require invasive treatment

### Follow-up outcomes

The median follow-up was 44.7 (range: 6–120) months and covered the 100% of study population. No aneurysm-related deaths were recorded. Ten deaths (10.6%) occurred during follow-up, three from cardiac causes, two from acute respiratory failure, three for coronavirus disease 2019-related pneumonia, one from cerebral ischemia and one from complications related to thoracic aortic surgery. The overall survival rate at median follow-up was 89.4%. At follow-up, freedom from aneurysm sac reperfusion or recanalization was observed in 89/97 (91.8%) cases. Aneurysm sac reperfusion was observed in 8/97 cases (8.2%), the majority of which represented residual sac perfusion. Two of them were characterized by residual sac perfusion of splenic aneurysms due to type II endoleak after sac-packing and sandwich-technique coil embolization, occurring at 67 and 6 months, respectively. Both were submitted to successful endovascular reintervention. The first was treated with additional coil embolization of the residual sac. In the second case, a superselective embolization of collateral branches from the gastroduodenal arcade supplying the aneurysmal sac was performed, followed by catheterization of pancreaticoduodenal collaterals reaching the sac. The residual sac embolization was then completed using a liquid embolic agent (Onyx™ 18–34, Medtronic, Irvine, CA, USA), which was never considered as first option for treatment in our Center. Two other sac reperfusions of renal VAAs after sac-packing coil embolization were treated by additional coil embolization of the residual sac at 60 and 24 months, respectively. The first subsequently underwent nephrectomy for a rapid increase in aneurysm size. Two patients treated for splenic and pancreaticoduodenal VAAs developed pseudoaneurysms distal to the previously placed devices. In the first case, the lesion occurred 6 months after emergency placement of a covered stent for splenic artery rupture. In the second case, a pseudoaneurysm developed 2 months after embolization with a vascular plug. Both the lesions were successfully corrected endovascularly. Two patients treated with sac-packing for hepatic and splenic VAAs developed partial sac reperfusion at 6 and 12 months, respectively. Both cases were left untreated due to stable aneurysm size and showed spontaneous regression by the 24-month follow-up. Freedom from aneurysm-related surgical and endovascular reintervention was achieved in 91/97 (93.8%) cases. The average diameter of the treated VAA at last follow-up was found significantly reduced compared to preoperative value (1.6 cm vs 2.93 cm; *p* < 0.001).

Long-term follow-up results, including overall survival, freedom from aneurysm-related complications, freedom from aneurysm sac reperfusion and reintervention were reported in Table 3.

**Fig. 3:**
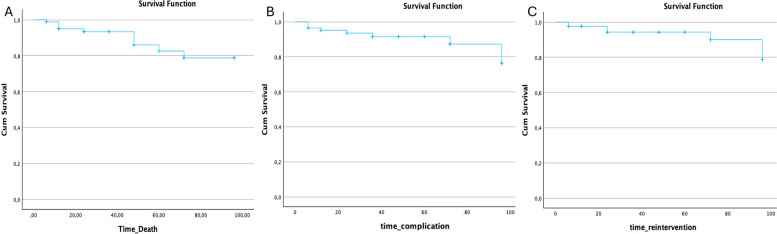
Kaplan–Meier survival curves for patients treated with endovascular therapy for true visceral artery aneurysms. **A** Overall survival over the follow-up period. **B** Freedom from aneurysm-related complications. **C** Freedom from reintervention. Censoring is indicated by crosses on the curves

Kaplan–Meier analysis of the cohort is shown in Fig. 3. Overall survival at 5 years was 79% (95% CI: 70–88%), while freedom from reintervention and aneurysm-related complications was 88% (95% CI: 81–95%) and 91% (95% CI: 85–97%), respectively.

## Discussion

Endovascular repair has progressively become the worldwide standard of care for the treatment of VAAs, offering high technical success rates and lower morbidity and mortality compared with open surgery [19–22]. In this single-center experience, we aimed to challenge the long-term performance of endovascular treatment for true VAAs in terms of efficacy, safety, and durability, by analyzing both our large real-world cohort and the expertise gained over a decade of practice.

The nature of the treated lesions is crucial when interpreting outcomes. Unlike several published series, we rigorously distinguished true aneurysms from pseudoaneurysms and included only true VAAs, given their different pathophysiology, clinical behavior, and management strategies [2, 3, 5, 8, 10, 19, 23, 24]. This approach yielded a relatively low proportion of ruptured aneurysms (16/97, 16.4% in our series) and reflects, together with the exclusion of pseudoaneurysms, the increasing use of high-resolution imaging and the consequent rise in incidental detection of small, asymptomatic lesions [12]. The trend towards elective treatment of such VAAs may improve rupture prevention but also raises concern about potential overtreatment; at the same time, accumulating operator expertise and technological advances may support earlier intervention in selected high-risk cases [2, 8, 9].

Treatment indications for VAAs remain limited by scarce high-level evidence and the absence of prospective comparative studies [25, 26]. Current recommendations are largely based on retrospective data and expert consensus, resulting in heterogeneous thresholds for intervention and follow-up across guidelines [3, 10, 11, 24, 27] The most recently updated ESVS 2025 Clinical Practice Guidelines provide more vessel- and location-specific recommendations and highlight that rupture risk may depend more on aneurysm location than size, particularly for pancreaticoduodenal and gastroduodenal aneurysms [10, 13].

Together with the Society for Vascular Surgery, the CIRSE Standards of Practice (2024) and the Italian SICVE–SIRM joint guidelines (2023), these documents promote multidisciplinary approach and endovascular repair as first-line therapy when anatomy is suitable [3, 11, 24]. Lower intervention thresholds are suggested for selected subgroups, such as women of childbearing age and patients with connective tissue disorders, reflecting a tendency towards earlier elective treatment in those at higher rupture risk [10, 13].

Beyond indications, prior studies and meta-analyses show that open and endovascular repair achieve comparable technical success and long-term survival, while endovascular treatment is associated with shorter hospital stay and at least similar reintervention rates [13, 28]. Current evidence also recommends CT angiography (CTA) or MR angiography (MRA) as preferred imaging modalities though surveillance intervals remain heterogeneous both for treated and conservatively managed VAAs [3, 10, 11, 29–31].

Within this framework, our results confirm that endovascular management meets expectations in terms of early and long-term outcomes. We observed a 100% technical success rate with no need for surgical conversion, slightly exceeding the 94–98% success reported in major European series such as Venturini et al. (technical success 97%, long-term reintervention 90%), Fargion et al. (technical success 94–98%), and Martinelli et al. (technical success 96%) [15, 20, 21]. No perioperative mortality occurred, and complications were infrequent, with 5.7% major and 13.4% minor short-term events, mainly limited and often asymptomatic organ ischemia. These outcomes align with CIRSE Standards of Practice and recent multicenter experiences [2, 12, 20]. At a median follow-up of approximately four years, no aneurysm-related deaths were recorded, and overall survival reached 89.4%, in line with contemporary literature [15, 20].

Freedom from aneurysm-related complications (92.8%), sac reperfusion or recanalization (91.8%), and reintervention (93.8%) in our series were comparable to or slightly higher than those published by Venturini et al., Fargion et al., and Martinelli et al., where freedom from reintervention ranged from 88–92% and freedom from aneurysm-related complications from 85–91% [12, 15, 20]. These findings support endovascular exclusion as a reliable long-term treatment strategy when coupled with structured imaging surveillance.

Procedural strategy was carefully tailored to aneurysm location, morphology, collateral circulation, and vessel characteristics [32–34]. Sac-packing embolization was mainly used for splenic and hepatic VAAs with a narrow neck, sandwich or isolation technique for more complex lesions involving bifurcations or tortuous segments, and covered stents or stent-assisted coiling for major trunks where distal perfusion had to be preserved. Importantly, devices with which operators had long-standing experience were favored over unfamiliar technologies, likely contributing to high technical success and safety [32–35].

This study has several limitations. Its retrospective, single-center design limits generalizability, particularly as it covers a considerable period during which treatment indications, techniques, and materials have evolved. The lack of a surgical control group and heterogeneity of aneurysm sites and techniques further limit result interpretation. Nevertheless, the large sample size, standardized imaging follow-up, and decade-long observation period strengthen the clinical relevance of these findings.

Overall, our findings suggest that endovascular management of true VAAs achieves its intended goals, providing a safe, effective, and durable treatment with excellent long-term outcomes.

Ongoing technological progress, advances in imaging, and growing operator experience further consolidate its role as the cornerstone of treatment for most patients, even if multidisciplinary decision-making remains crucial to ensure optimal patient selection and individualized procedural planning.

The increasing detection of small, asymptomatic VAAs may further expand the preventive role of interventional radiology. Future subgroup analyses, such as correlations between clinical presentation, aneurysm characteristics, and selected techniques, could therefore provide additional insights to refine patient-tailored strategies and optimize outcomes.

## Data Availability

The datasets generated during the current study are available from the corresponding author on reasonable request.
